# Assessment of the interstitial volume in healthy volunteers. An equilibrium contrast CMR study

**DOI:** 10.1186/1532-429X-14-S1-P222

**Published:** 2012-02-01

**Authors:** Daniel Sado, Viviana Maestrini, Andrew Flett, Steven K White, Sanjay M Banypersad, Jonathan Hasleton, Graham Sado, James Moon

**Affiliations:** 1 Imaging Centre, The Heart Hospital, London, UK; 2 General Practice, The Enterprise Practice, London, UK

## Summary

In this study we have used Equilibrium contrast CMR (EQ-CMR) to evaluate the cardiac insterstium in healthy volunteers creating a table of normal values and finding no correlation with age, but differences with gender, height and hematocrit.

## Background

EQ-CMR is a new method for assessment of the myocardial contrast volume of distribution, Vd(m), a marker of interstitial space. We used EQ-CMR to assess the Vd(m) in healthy volunteers to ascertain normal age/gender values.

## Methods

86 healthy subjects (median age 45, range 24-81, 42 males) were recruited from hospital, university or via a local General Practitioner surgery. All were assessed with a cardiac history and examination, routine blood tests, 12 lead ECG, blood pressure and clinical CMR. Volunteers were excluded if any evidence of cardiovascular disease was found.

Baseline differences between male and female volunteers were found, with the latter having a faster heart rate, smaller left ventricular volumes, shorter height, lower weight and lower hematocrit.

## Results

**Figure 1:**
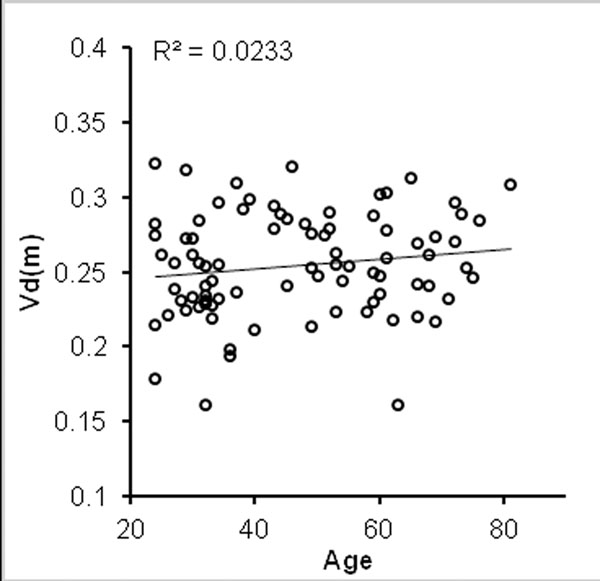


**Figure 2:**
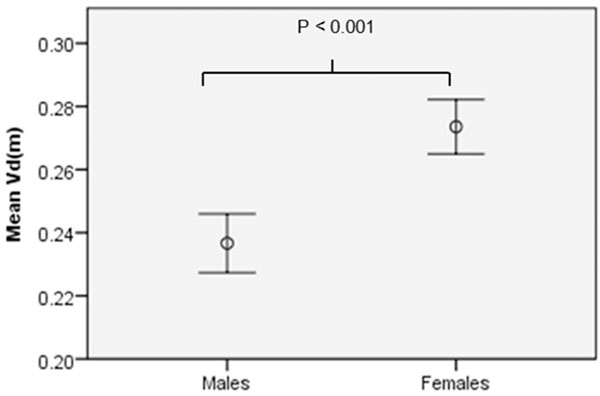


Mean Vd(m) was 0.255±0.035. There was no significant correlation between age and Vd(m) (R=0.15, P=0.16, Fig 1). Vd(m) in females was significantly higher than males (0.275±0.028 vs 0.237±0.031, P<0.001) Fig 2). Negative correlations were found between Vd(m) and height (R=-0.41, P<0.001), left ventricular mass (-0.38, P<0.001) and hematocrit (R=-0.41 P<0.001) - this last variable in females but not males.

## Conclusions

Vd(m) did not change with age, but did with gender, height and hematocrit. Several plausible biological explanations exist for this gender finding, and support for the lack of age related changes exists in two large human necropsy studies. Further work will be required to evaluate these findings further.

## Funding

1) British Heart Foundation

2) GlaxoSmithKline

